# Health Care Professionals’ and Patients’ Perceptions and Experiences of Who Uses Video Consultations, and Why: Qualitative Study

**DOI:** 10.2196/68658

**Published:** 2026-03-05

**Authors:** Irene Muli, Åsa Cajander, Nadia Davoody, Lovisa Jäderlund Hagstedt, Helena Hvitfeldt, Maria Hägglund, Marina Taloyan

**Affiliations:** 1 Participatory eHealth and Health Data Research Group, Department of Women’s and Children’s Health, Uppsala University, Dag Hammarskjölds väg 14B, Uppsala, 75237, Sweden; 2 Department of Information Technology, Uppsala University, Uppsala, Sweden; 3 Department of Learning Informatics, Health Informatics Centre, Management and Ethics, Karolinska Institutet, Stockholm, Sweden; 4 Norrtälje Hospital, Vårdbolaget Tiohundra, Stockholm, Sweden; 5 Uppsala University Hospital, Uppsala, Sweden; 6 Academic Primary Healthcare Centre, Region Stockholm, Stockholm, Sweden; 7 Department of Neurobiology, Care Sciences and Society, Karolinska Institutet, Stockholm, Sweden; 8 Tbilisi State Medical University, Tbilisi, Georgia

**Keywords:** video consultations, primary care, introduction, perceptions, drivers, Sweden

## Abstract

**Background:**

Video consultations (VCs) are effective and beneficial, yet their use is being discontinued, and there is a preference for face-to-face consultations.

**Objective:**

This study investigates how patients and health care professionals (HCPs) perceive patients’ introduction to VCs, who use them, and what drives their use in Swedish primary care.

**Methods:**

Six focus group interviews with 27 HCPs and 13 individual interviews with patients in primary care were conducted between August 2022 and May 2023. The interviews examined VC implementation and were analyzed using rapid assessment procedures.

**Results:**

A total of five themes were identified: (1) challenging start with unprepared users and immature technology; (2) users and nonusers are perceived to have different characteristics, needs, and circumstances; (3) patient-related drivers: based on patients’ preferences and opportunities; (4) HCP-related drivers: clinical suitability, assessment of patient needs, and preferences; and (5) societal and organizational drivers: the pandemic, demographics, and infrastructure. Patients and HCPs described the introduction of VCs as rushed and confusing, with limited guidance and support (theme 1). HCPs struggled to assist patients due to a lack of training and limited access to the patient-facing interface (theme 1). VC users were typically perceived as younger, digitally literate, and motivated by convenience or urgency, while older adults and those with language or cognitive barriers were often assumed to be nonusers (theme 2). VC use was shaped by patient preferences, accessibility, and clinical urgency (theme 3), as well as by HCPs’ professional judgment and convenience (theme 4). Assumptions held by HCPs about patients’ digital skills and preferences influenced whether VCs were offered, while patients’ own assumptions about complexity or suitability affected whether they accepted them. Broader factors, such as digital infrastructure, platform usability, reimbursement policies, and the COVID-19 pandemic, also significantly influenced use (theme 5).

**Conclusions:**

The rushed implementation potentially deterred some patients and HCPs from use. Misguided preconceptions and biases negatively influenced VC use and risked reinforcing existing disparities and contributing to digital exclusion. In addition, HCPs’ and patients’ preferences, which were related to their needs, waiting times, and different circumstances, and potentially misguided judgments of appropriateness, influenced VC use. Lastly, infrastructure, reimbursement, sociodemographics, and organizational type also drive VC use. To support more sustainable and equitable use of VC in primary care, developers should optimize VC applications’ usability, implementers should deploy multiple strategies, health care providers should consider the potential of VC in care delivery, and policymakers should increase digital readiness. Further research should evaluate the effectiveness of different strategies for introducing patients to VCs, explore younger patients’ and nonusers’ perspectives, characteristics of HCP users, and differences between professional roles, as well as between consultation types.

## Introduction

### Background

The rapid increase in video consultations (VCs) is an example of the accelerating digitalization of health care [1,2]. However, despite this progress, some studies indicate that VC use, which peaked during the COVID-19 pandemic, has since declined [3-5].

Teleconsultations, such as VCs, are generally as effective as face-to-face consultations regarding clinical outcomes in primary care and mental health [6] and could, in some cases, be a superior alternative to telephone consultations (TCs) [7]. Additionally, both patients and health care professionals (HCPs) have reported high satisfaction with VCs [6,8-10]. Despite these positive findings, some patients and HCPs still prefer face-to-face consultations [9-11].

The way that the use of VCs is implemented might explain the discontinuation of VC use and preference for face-to-face consultations [12]. However, research on implementation is limited to only a few regions or countries and secondary care settings [13-15], with studies mainly investigating HCPs’ experiences and organizational challenges [13]. While existing studies often focus on postimplementation satisfaction, they tend to overlook the implementation process, particularly from the patients’ perspectives [16,17]. When patient introduction to VCs is described, it is often vague and lacks sufficient detail [18,19]. No studies have been found that conduct an in-depth investigation of patients’ introduction to VCs.

In the research project ePrIm (implementation of eHealth in primary care), the overall aim was to explore how the implementation of eHealth in Swedish primary care affects the work environment of HCPs and patient satisfaction with care. The implementation of VCs was 1 of 3 case studies conducted in the project, the other 2 focusing on patients’ online record access [20] and digital self-management of asthma. In this study, we focus on results from the VC case study, specifically on patients’ introduction to VCs and perceptions of who may or may not want to use VCs.

Perceptions of who the users and nonusers of VCs are might influence VC use [21]. Although there is considerable research on the perceptions of VCs, little is known about the perceptions of who the users and nonusers of VCs are. In the context of this study, “perceptions” comprise the attitudes, beliefs, and subjective judgments held by HCPs and patients about VCs. This includes how they view the characteristics, needs, and potential use of VCs for different patient groups, as well as their opinions regarding the suitability of VCs for various medical scenarios. Further, few studies have explored the drivers of VC use and nonuse in-depth [21-23]; drivers in this study refer to factors influencing use either positively or negatively.

This study addresses notable gaps in the current research: patients’ introduction to VCs, perceptions of users and nonusers, and the drivers of VC use, in a Swedish context. By collecting data from patients and HCPs, this study offers a comprehensive perspective on the factors influencing VC adoption and use. This dual approach is valuable because it allows for a holistic understanding of the potentially conflicting motivations, experiences, and barriers perceived by the 2 groups. By examining both sides, this study can identify discrepancies and areas of potential improvement, providing a richer and more nuanced understanding of VC use. Given the reported benefits of VCs for patients, HCPs, and society, it is vital to expand our understanding of why there is a risk of discontinuing their use in various contexts and why there remains a preference for face-to-face consultations.

### Aim

This study aimed to investigate the dual perspectives of patients and HCPs on patients’ introduction to VCs, perceptions of VCs users, and drivers of use in Swedish primary care. More specifically, this study investigated: (1) HCPs’ and patients’ experiences of patients’ introduction to VC, (2) HCPs’ and patients’ perceptions of who uses or does not use VC, and (3) HCPs’ and patients’ experiences and perceptions of drivers of use or nonuse of VC.

## Methods

### Study Design

This study had a qualitative approach, using focus groups and individual interviews to capture the experiences and perceptions of HCPs and patients on the introduction and use of VCs.

### Study Setting

Digitalization of health care services is widespread in Sweden. According to a national assessment, digital consultations (ie, consultations using applications that replace in-person consultations, eg, VCs) with physicians have increased from 10% in 2019 to 24% in 2022 [24,25]. Nearly all Swedes have internet access at home, and over 90% use it daily [24].

This study was conducted in Region Stockholm, 1 of 21 regions responsible for providing tax-funded health care in Sweden [26]. Tax-funded health care can be either publicly or privately managed, and a third of the care in Region Stockholm is privately managed [27]. This study involved both privately and publicly managed health care centers. The care fees are the same within the region but will vary depending on the patient’s citizenship [28]. VCs cost the same as face-to-face consultations for the patient, although the care provider’s reimbursement is lower for VCs [29].

The region’s over 200 primary care centers (PCCs) and family doctor offices are the gateway to health care from which referrals to specialized care are made when necessary [28] PCCs typically comprise a mix of HCPs.

Initially, direct-to-patient VCs were provided through online platforms and applications developed and managed by private health care providers [30]. Progressively, especially during the COVID-19 pandemic, platforms and applications have emerged in the public sector. *Alltid öppet* [always open] is such a platform, which was developed by the public health care provider Region Stockholm in 2018. *Alltid öppet* supports both VCs and consultations through chats between patients and HCPs and currently (2024) has 2 million users. PCCs can choose which digital services they offer through the platform, and, in some cases, this includes online appointment booking by the patients themselves. Parents can use the application on behalf of children younger than 13 years of age. Use requires authorization with electronic identification (eID). *Alltid öppet* was the VC service used by both HCPs and patients in this study.

### Recruitment of Study Participants

All participants were recruited from 10 PCCs in Region Stockholm; 5 rural PCCs in the most northern part of Stockholm (Norrtälje), and 5 more centrally located PCCs with a variation in sociodemographic contexts (Jakobsberg, Hässelby, Liljeholmen, Gustavsberg, and Huddinge PCC).

All HCPs from the participating health care centers with experience in conducting VCs through *Alltid öppet were* invited to participate. All eligible HCPs were sent invitations via email with information about this study and were followed up with help from the PCC manager, who helped schedule the focus groups at times convenient for the volunteering HCPs. The participating HCPs also received information about this study before the interview. The focus groups consisted of HCPs of different professions: general practitioners (GPs), nurses, counselors, psychologists, and physiotherapists. The HCPs were also diverse with regard to their experience of using VCs with patients and as patients themselves. A total of 6 focus group interviews were conducted with 3 to 6 participants in each; in total, 27 HCPs participated. All but 1 focus group consisted of HCPs from the same PCC; the remaining group consisted of participants from 2 smaller PCCs.

Patients were recruited to the current study through a previously conducted survey about VC use through *Alltid öppet* [11]. Survey participants were eligible to participate if they were 16 years or older and had a VC through the *Alltid öppet* application from March to May 2022. Invitations to participate in the survey study were sent via text message. After completing the survey, respondents were invited to participate in the interview study. Those interested in participating received a link to a short questionnaire, separate from the survey, which also provided further information about the interview study. The questionnaire collected information regarding: contact details, demographics, and frequency of VC usage. A diverse group of respondents (Table 1) was then contacted via telephone and invited to participate. All participants received full study information; consent to participate and record the interviews was obtained before the interview. In total, 13 patients were interviewed.

**Table 1.** Characteristics of the interviewed patients, Region Stockholm, 2022.

Patient characteristics	Values
Sex	
Female	7
Male	5
Other or do not want to answer	1
Age (years)	
25‐34	2
35-44	3
45-54	1
55-64	4
65-74	2
75-84	1
Highest education level	
Primary school	1
High school	3
Adult education [29]	1
University or university college <3 years	2
University or university college >2 years	6
Total number of VCs^a^ via Alltid öppet^b^	
1-5	7
6-10	2
More than 10	1

a VC: video consultations.

b Alltid öppet: ”always open”; an application for video consultations.

### Data Collection

Data were collected using focus group interviews with HCPs and individual semistructured interviews with patients. The focus group interviews were conducted between December 2022 and May 2023, and the patient interviews between August and December 2022. The dates were determined by the project plan and the participants’ availability. The choice to use focus group interviews with HCPs and individual semistructured interviews with patients was motivated by the desire to capture the collective dynamics and shared experiences among HCPs in a group setting, while allowing for a more in-depth exploration of personal experiences and perceptions in the one-on-one patient interviews. Both the focus groups and the individual interviews were audio-recorded and transcribed verbatim.

All focus groups, except one, which was performed in person, were conducted through videoconferencing at their workplace. A semistructured interview guide was used in the focus group interviews. The guide was based on the overall project aim to investigate the implementation of VCs through *Alltid öppet*, its impact on the work environment and work processes, as well as the HCPs’ perceptions of patients’ introduction to and use of VCs. In this study, our focus is on the latter. Due to our limited access to HCPs’ time, we chose not to pilot the focus group interview guide but instead critically assessed it after each focus group to ensure all important aspects were covered. The interviews were 45‐60 minutes long and, because they were conducted during the HCPs’ lunch break, lunch was provided as a token of appreciation.

The interviews with patients were conducted via telephone or videoconferencing, based on participant preference. The semistructured interview guide used in the interviews was pilot-tested, leading to minor changes. The interview guide was based on the overall project aim to investigate how and why patients use VCs and included questions about patients’ introduction to VCs and factors influencing VC use. The interviews were, on average, 30 minutes long and were conducted until data saturation was reached, at which point no new information emerged. After the interview, participants received a gift card as a token of appreciation.

The focus group interviews with HCPs were conducted by MH, the moderator, with at least 1 facilitator. IM, LJH, ND, and MT facilitated at least 1 focus group interview each. The facilitators assisted the discussions by asking follow-up questions or providing the moderator with questions to ask, and by observing nonverbal communication. IM conducted the individual patient interviews. The moderator and the facilitators were all female. MH is a researcher or lecturer with a PhD in medical informatics, IM and LJH were both PhD students in digital health. LJH is also a practicing medical doctor who works at one of the PCCs included in this study but did not facilitate the focus group interview with HCPs from her PCC. ND also has a PhD in medical informatics, and MT has a PhD in medical science. MH, who moderated the focus group interviews, and IM, who conducted the individual interviews, had extensive knowledge and experience in qualitative research at the time of the interviews. MT, who, together with IM, conducted the analysis, also had extensive knowledge and experience in qualitative research. To minimize potential bias and maintain a neutral interview environment, the researchers did not disclose any personal goals or motivations related to this study to participants.

While data saturation was reached for patient interviews, it was not fully achieved for HCPs due to practical constraints. Due to HCPs’ busy schedules, many were unable to participate, and for those who could, interviews were conducted only during their lunch breaks.

### Analysis

Both the focus groups and individual interviews were analyzed using rapid assessment procedures (RAP) [31]. RAP was informed by the time-sensitive nature of the broader project and the need for structured yet efficient analysis. The broader project’s different parts were conducted simultaneously, and as is often the case with research, the resources were limited. To conduct all the studies and meet the strict deadline, we had to choose methods that offered efficiency without compromising rigor. RAP allows for a systematic and flexible approach to analyzing qualitative data, facilitating the rapid identification and synthesis of key themes and patterns without compromising the depth and reliability of the findings. RAP has been successfully used in health care research, particularly in implementation and evaluation studies where timely insights are crucial [32,33]. RAP was thus selected because it is an established qualitative approach that enables efficient yet rigorous data analysis; in other words, it does not compromise depth when properly executed. To support consistency and reliability in this study, we used structured templates, independently piloted the coding approach, and held regular analytic discussions to align interpretations across researchers.

This study follows an exploratory qualitative design, allowing themes to emerge organically from the data rather than fitting them into a predetermined theoretical model.

Following the RAP methodology, the first step involved creating neutral domain-based templates based on this study’s aim. For step 2, the templates were independently piloted with 1 interview by IM and MT. In step 3, the analysis and usability of the template were discussed to ensure intercoder reliability, and the domains were clarified and further piloted. Of step 4, when consistency in analysis was reached, the remaining interviews were divided between IM and MT, who summarized vital points. The final domains in the RAP template were (1) patients’ introduction to VC, (2) perceptions of patient users and nonusers, and (3) drivers of patients’ use and nonuse. In step 5, emerging key points were discussed and categorized, after which categories were grouped into themes. We recognize that perceptions of patient users and nonusers and drivers of patient use and nonuse overlap to some degree; however, we believe that there is a distinction that is worth exploring further. IM used NVivo (version 1.4; Lumivero) and MT used pen and paper.

This study was reported in accordance with the COREQ (Consolidated Criteria for Reporting Qualitative Research) [34]. This study used the generative artificial intelligence technology Grammarly (Superhuman Platform) to enhance the grammatical accuracy and vocabulary richness.

### Ethical Considerations

This study received ethical approval from the Swedish Ethical Review Authority (reference number 2021‐05096). All study participants provided written or oral informed consent before participating in this study. In accordance with the Swedish Ethical Review Authorities’ requirements, participants were informed that their participation was voluntary and that they could choose to revoke their participation at any time. Transcribed interviews and focus groups were deidentified to protect the participants’ privacy, and confidentiality was preserved when reporting results. In accordance with the ethical approval, patients were compensated with a gift certificate (200 SEK, equivalent to US $20), whereas HCPs received lunch during the focus group interviews.

## Results

### Overview

**Figure 1.**
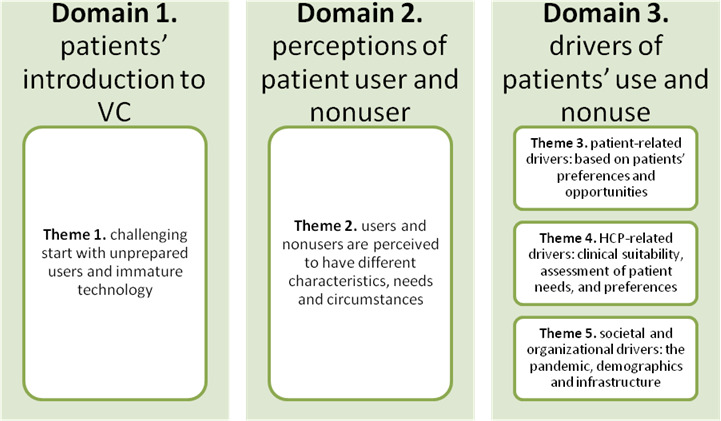
The identified themes under the 3 predefined domains, rapid assessment procedures, patients, and health care professionals, Region Stockholm, 2022‐2023. HCP: health care professional; VC: video consultation.

A total of five themes were identified under the three predefined domains: (1) patients’ introduction to VC, (2) perceptions of patients as users and nonusers, and (3) drivers of patients’ use and nonuse. The first and second domains had 1 theme each, while the third had 3 themes (Figure 1).

### Domain 1. Patients’ Introduction to VC

#### Theme 1. Challenging Start With Unprepared Users and Immature Technology

In this study, the patients’ introduction refers to all activities aiming to inform patients about the service and help them start using it.

Patients’ introduction to VCs encountered many challenges. On the one hand, some patients had acquired the necessary eID and accessed the application due to the COVID-19 pandemic, using it to schedule COVID-19 tests and vaccinations, which facilitated the introduction of VCs. On the other hand, the need to promptly implement VCs due to the pandemic left little room for a structured introduction of VCs.

According to HCPs, patients were provided with brochures and instruction videos online but needed guidance to find them. With the invitation to a VC, patients were also provided with instructions for downloading the application and could receive additional assistance with the download, if required, from HCPs, either over the phone or physically at the PCC. Unforeseen issues occurred during the installation of the VC application, which HCPs had to assist patients with. Significant guidance was provided over the phone in the beginning. Assisting patients in using the services was challenging for HCPs because they lacked manuals and were themselves unfamiliar with the service. They also lacked access to the patients’ user interface, which made helping patients navigate the VC tool difficult. Communicating with patients with language barriers and instructing older adults with physical impairments, such as poor eyesight, had also been challenging. According to HCPs, informal caregivers or relatives were important in assisting with the use and scheduling of VCs.

HCPs also expressed that there was some resistance from patients who did not want to acquire the necessary eID; the patients did not trust it due to reports of related fraud in the media. This was experienced as challenging by HCPs because they were not able to help those without an eID, and explaining this to patients was time-consuming. Getting started was particularly challenging for older patients, according to HCPs, presumably due to low digital literacy. Older adults were stressed and worried about technical difficulties. According to HCPs, patients were inexperienced, confused, and often became disconnected due to a lack of knowledge, leading to many planned VCs being converted to TCs. HCPs tried to find what worked best from case to case. Some of this confusion was believed to be related to the application’s usability. The service itself was also described as unstable.

*There was a lot of calling back and forth in the beginning since patients couldn’t connect or didn’t know how to.*

[FG 1]

*The application or the video feature had many bugs initially, it could…I mean, half of the consultations worked, and half didn’t.*

[FG 2]

These reported issues improved over time as both patients and HCPs became more experienced, and the application itself was updated and became more stable.

Patients’ experiences aligned with those of the HCPs. They initially experienced technical difficulties and uncertainties at both ends. Although the introduction was confusing for some patients, they were familiar with the technical difficulties, which was something they had expected. According to patients, they received information from HCPs, found information themselves, or coincidentally found out about the service.

*[…] then they had long waiting times, so they said; “Would you consider having a VC?*” *Yes, that works…I wasn’t aware it was possible to book a VC.*

[P1]

### Domain 2. Perceptions of Patient User and Nonuser

#### Theme 2. Users and Nonusers are Perceived to have Different Characteristics, Needs, and Circumstances

In this study, “perceptions” refers to the attitudes, beliefs, and subjective judgments held by HCPs and patients about patient users of VCs.

According to HCPs, patient users of VCs were usually young. Users were perceived to have a positive attitude toward VCs and varied needs. They saw the benefit of VCs, wanted contact urgently, had minor complaints, or were on sick leave. Patient users were almost always follow-up patients who did not need to come to the PCC. People with psychosocial needs were also common users. Further, patient users of VCs were perceived to have special circumstances. They usually had longer geographical distances to the PCC, worked full-time, or experienced other obstacles or barriers to physically attending a meeting at the PCC.

*Those with social phobia thought VCs were great.*

[FG 3]

According to HCPs, users were perceived to have certain capabilities; they were those who scheduled a VC themselves and those who often had less serious health care needs. HCPs expressed that by doing so, they used resources that should be allocated to patients with greater needs, something they perceived as unfair.

In contrast, according to HCPs, nonusers were usually older, although not all older patients were nonusers. Nonusers usually preferred or required face-to-face visits.

Patients also perceived VCs to be great for people struggling with mental health issues, for example, social anxiety, which prevented them from seeking care in person at the PCC. Moreover, patients believed that older people lacking digital literacy were nonusers. Having at least some digital literacy or experience of internet use was seen as a prerequisite to using VCs in order to be comfortable with them. VCs were also considered to exclude people lacking technical and language skills.

*I think a large proportion of patients are excluded. Partly because they can’t handle the technology, and then there is the language barrier.*

[P2]

### Domain 3. Drivers of Patients’ Use and Nonuse

#### Theme 3. Patient-Related Drivers: Based on Patients’ Preferences and Opportunities

Drivers in this study refer to factors influencing use either positively or negatively.

According to HCPs, the use of VCs was significantly based on patients’ preferences. The majority of patients requested face-to-face meetings or TCs. These requests were assumed to be due to familiarity with face-to-face consultations and the need for contact. On the other hand, some VCs were scheduled at patients’ requests, and patients sometimes requested a VC when HCPs would have preferred a face-to-face meeting. Moreover, some HCPs always checked the patient’s ability to have a VC even when they thought VCs were appropriate.

*For those scheduled for a VC, it’s my impression that it’s often the patient who requested it for some reason.*

[FG 4]

Impatience or need for immediate contact was another factor influencing VC use, according to HCPs. Due to long waiting times for face-to-face consultations, a VC could be chosen in order to see an HCP sooner. According to HCPs, quick contact or the ability to have a consultation instead of rescheduling due to mild but contagious illnesses were influential factors for preferring VCs. Having sick children could be a factor influencing their caregivers’ use of VC. Being on sick leave or geographical distance to the PCC were not only common characteristics of users, but also acted as drivers of VC use. Patients who had moved may want to remain in contact with a health care provider with whom they had an established relationship, ensuring continuity of care. Difficulties patients experienced in booking a VC were identified by HCPs as a factor negatively influencing use.

According to HCPs, immigrants, older persons, and teenagers are, in some instances, unable to acquire an eID. Moreover, some people could be constrained by law, for example, due to conservatorship, where they are unable to make their own decisions. Other patients simply did not wish to have eIDs, according to HCPs; as previously mentioned, they did not trust this due to reports of related fraud in the media.

Some patients expressed that VCs felt impersonal compared to a consultation at the PCC, saw no need to have VCs, and preferred face-to-face and TCs for inquiries. Others preferred face-to-face as an initial contact, while VCs were more appropriate for follow-up visits, while others thought that VCs could be used for the initial assessment. The ability to make the necessary assessments would also influence use, according to the patients, as would the type of care needed and their ability to reach the PCC. Patients thought VCs worked well when avoiding infections and when they were at home with children. Additionally, the opportunity to meet with a physician and the ability to have quick contact were factors that influenced use.

*I think it’s a great compliment, especially if you’re like me. I’m quite sensitive to infections, so instead of scheduling a meeting when you have a cold or are at home with a child with a cold, you could schedule a VC.*

[P3]

### Theme 4. HCP-Related Drivers: Clinical Suitability, Assessment of Patient Needs, and Preferences

When deciding on whether to offer a VC or not, HCPs focused on the patient’s complaint, what type of care this would require, their assessment of the patient’s needs, and what combinations of these factors made a VC suitable. Making these assessments had been learned over time, as there were no clinical guidelines they could follow.

While there are some routines surrounding infections, in cases where in-person assessments and investigations were required and where VCs would be inappropriate, most decisions of whether or not to use VCs were still based on the individual HCP’s assessment. According to some HCPs, VCs were potentially inappropriate for mental health patients needing cognitive behavior therapy. VCs were also perceived as inappropriate in cases where communication could be challenging, for example, patients with cognitive impairment, or severe autism, and for complex patient cases, for example, patients with co-morbidities or very old patients. There was also an initial assumption that VCs would not work well for older patients, and while some HCPs expressed that this perception had changed over time, for others, this assumption was still a driver of VC use.

*People with multiple illnesses, maybe older individuals. That’s a patient group to which I, maybe wrongly, don’t offer VCs.*

[FG 5]

When physical assessments were not necessary, use was based on patients’ needs. Patients’ safety and integrity during a VC were considered important factors. If there were concerns for patients’ safety or integrity during a VC, HCPs might choose to schedule a follow-up face-to-face consultation at the PCC instead of a VC. Moreover, third parties involved in the consultation, for example, translators, could further complicate the use of VCs, but the technology could also facilitate participation of, for example, sign language interpreters. HCPs also considered VCs functional for follow-ups and saw a time advantage in having shorter VCs, potentially releasing time to serve more seriously ill patients at the PCC.

Furthermore, VC use was based on HCPs’ preferences and convenience. HCPs preferred a face-to-face first encounter with patients. Some HCPs only offered their patients a face-to-face visit, and others offered VCs, sometimes without an option to choose a face-to-face visit. Physicians doing their residency would also opt for face-to-face appointments for educational reasons. Some HCPs would schedule VCs when they worked from home or elsewhere, for example, due to mild infectious diseases, thereby avoiding sick leave and rescheduling of appointments. Further, some HCPs opted for TCs due to initial technical issues. Technical limitations could also affect their assessment, for example, making VCs suboptimal for skin conditions due to the low image resolution.

According to patients, continuity of care, that is, being able to have a VC with a familiar HCP, influenced their use. Patients relied on the HCPs’ judgment, and HCPs’ recommendations influenced their use of VCs. Further, 1 patient expressed a willingness to consider VCs if it benefited HCPs and their work environment.

### Theme 5. Societal and Organizational Drivers: the Pandemic, Demographics, and Infrastructure

According to HCPs, the COVID-19 pandemic greatly influenced and accelerated VC use for both patients and HCPs. Social distancing was a catalyst for HCPs scheduling VCs to reduce the number of patients in the waiting room.

Sociodemographic factors were other influential factors according to HCPs. They believed that having younger and older patients unable to use VCs, for example, due to a lack of necessary software, might have influenced overall VC usage. Moreover, internet connectivity, or lack thereof, for example, in rural areas, was an issue that greatly influenced VC use. Changes to the software that enable VCs, and various updates, influenced the ability to conduct VCs. Seasonal differences had also been observed, with patients requesting VCs more during the winter and summer.

*It feels like everyone wants digital [consultations] during summer or winter, but in spring and autumn, which are working seasons, they come in [to the PCC] more.*

[FG]

HCPs expressed that the lower threshold for having a VC compared to a physical appointment at the PCC had led to an increase in overall appointments. Another influencing factor was the reimbursement system, which included fees and compensation for VCs but not for TCs. HCPs felt guilty about making patients pay for issues that could be resolved through a phone call, yet felt pressured to do so to increase PCC resources. Moreover, existing work processes also influenced the use of VCs. Nurses were sometimes unable to have VCs during emergency hours because they needed to be able to respond to potential emergencies at the PCC. Furthermore, VCs were viewed not as a replacement but rather as a complement to physical care.

Patients confirmed the influence of the pandemic on their use of VCs. For some patients, being able to use a public sector application for VCs was also important.

## Discussion

### Principal Findings

This is one of the few studies investigating patients’ introduction to VCs, perceptions of VCs’ users, and drivers of use in primary care. While the COVID-19 pandemic forced a rapid introduction of VCs into Swedish primary care, positively influencing VC use, the rushed, unstructured process might also have negatively affected VC use. Perceptions and preconceptions about users and nonusers were also influential. Among other drivers of VC use were both HCPs’ and patients’ preferences and judgments of appropriateness, as well as several broader organizational and societal factors.

### Pandemic, HCPs, and Preconceptions Influence VC Use

One of the key findings of this study was that the COVID-19 pandemic and patients’ introduction to VCs both positively and negatively influenced VC use. The pandemic’s influence aligns with earlier research [17,23,35]. The pandemic positively influenced VC use by facilitating its introduction and accelerating its use. The pandemic, however, also rushed implementation, contributing to a challenging start. The technology was immature, and patients and HCPs were unprepared, which also highlighted broader issues of societal and organizational readiness for eHealth adoption. The extraordinary circumstances may have increased openness to digital solutions and partially offset the effects of a suboptimal introduction. However, the hurried implementation may have led to poor user experiences, undermining trust and satisfaction with the service. These early frustrations may also have shaped long-term attitudes toward VCs, deterring some patients and HCPs from continued use. Under normal circumstances, a more structured rollout, one that included efforts to understand and address patients’ concerns, might have improved acceptance and usability. However, given the urgency of the pandemic, such user-centered planning was likely unfeasible.

Another key finding of this study was how important HCPs were in the introduction of VCs. Most patients were introduced to the service by HCPs [36]. However, the lack of preparedness among HCPs contributed to many of the challenges they experienced. This issue has also been expressed by other HCPs, where initial usability and technical issues were attributed to the lack of training of some staff [18,37].

Additionally, HCPs’ preconceptions influenced VC use. Several HCPs admitted being less likely to suggest VCs to older patients, based on assumptions about digital literacy and willingness to engage with technology. Although some later recognized that age alone was not a reliable indicator of VC use, these assumptions likely shaped access to digital services in practice. These findings raise significant concerns about equity and inclusion in digital health care. When HCPs make assumptions about patients’ abilities or preferences, particularly based on age, disability, language skills, or other perceived vulnerabilities, they risk reinforcing existing disparities and contributing to digital exclusion. Previous research indicates that most people with disabilities have an overall positive experience of VCs [38]. Additionally, although they encountered difficulties, patients who faced language barriers also reported high satisfaction with VC services [39].

Notably, the role of HCPs extended beyond simply offering or withholding VCs. Patients often reported relying heavily on HCPs’ recommendations when deciding whether to accept a VC, underscoring the strong influence of provider endorsement. This dynamic aligns with prior research demonstrating the significant impact of HCPs on patients’ willingness to adopt digital services [40].

In contrast, there is also reason to believe that bias regarding older adults and technology use exists among patients themselves. Several participants perceived older individuals as less likely to use VCs, reflecting common stereotypes around age and digital competence. This perception aligns with the concept of effort expectancy, a key factor influencing both the intention to use and actual adoption of technology [40], which may be negatively affected by assumptions that older adults lack digital literacy or confidence. If older patients expect VCs to be difficult or complex, these beliefs can undermine their self-efficacy and willingness to engage, thus reducing actual uptake.

Moreover, the initial challenges in introducing VCs in this case, particularly for older users, may have reinforced these negative perceptions, contributing to a cycle of continued nonuse or disrupted engagement. This suggests that both individual and systemic factors may interact to maintain digital exclusion among older populations. Without proactive efforts to support digital confidence and ensure accessible, user-friendly design, such exclusion risks becoming self-perpetuating.

In addition to individual perceptions, broader concerns about fairness also emerged as a factor potentially limiting the adoption of VCs. Some participants expressed the view that VCs might unfairly disadvantage certain patient groups, particularly those with more complex care needs, a concern echoed in earlier studies [17,41,42]. If VCs are perceived as excluding vulnerable or high-need populations, this could undermine their perceived value and legitimacy as a mode of care delivery more generally.

To summarize, the pandemic, HCPs, and preconceptions about and among patients influenced VC use both positively and negatively.

### Preferences Influence Use and Are Related to Needs, Waiting Times, and Different Circumstances

Another key finding of this study was that VC use seemed to be greatly influenced by HCPs’ and patients’ preferences, which in turn were influenced by a range of different factors.

In our study, patients’ preference for face-to-face consultations was related to their need for physical contact, which has been previously highlighted [17]. This could be related to the quality of VCs, where VCs do not fully emulate face-to-face interactions. Familiarity with face-to-face interaction was also thought to be related to its preference. To some extent, therefore, preference could be a matter of exposure and experience. The dynamic of an in-person meeting can also differ from a virtual one, negatively affecting the VC experience and preference. HCPs have, for example, reported less prepared patients who might try to multitask during the VC [30,37].

Challenges with waiting times within Swedish health care seem to be another significant driver of preference and VC use, according to both HCPs and patients. While VCs are offered and used for quicker contact, the waiting time could also drive inappropriate use of VCs. If VCs are chosen for their shorter waiting times, rather than their clinical appropriateness, this might negatively influence experiences and the intention to continue using VCs [11]. Moreover, as previously highlighted, VCs could reduce waiting times [37,43], whereas shorter VCs for simpler cases could allow time for severe cases. Since VCs could be offered as an alternative to rescheduling, a visit could reduce treatment delays.

Other factors that appear to influence preferences include geographical distance, seasonal factors or weather conditions, and health in general. Geographical distance could be related to accessibility for people in rural areas and those with busy schedules or mobility issues [23]. Weather conditions could also be related to mobility issues and comfort; for example, winter storms can hinder travel. It could also be related to conditions during these seasons, such as the increase in flu infections in the winter and allergies in the summer, where examinations and in-person consultations might be unnecessary. Canadian HCPs have reported that virtual visits enable them to provide care during bad weather conditions [37]. This highlights the potential for VCs to address some of the seasonally related care delivery challenges, which could be considered during resource planning.

The ability of HCPs to choose when to have VCs offers control over their work and flexibility, which has previously been reported [35], and might positively influence their work environment, health, and preferences. However, technical issues could negatively influence their preferences and their perceived patient preferences.

Additionally, VCs have also previously been viewed as complementary due to a lack of research and quality, and usability issues have been reported as causing the preference for telephone or face-to-face consultations over VCs [9,23,37]. Further, the reports of patients requesting VCs could be evidence of an overassumption that most patients want or prefer a face-to-face consultation.

In a nutshell, personal preferences for VC or a face-to-face consultation, which heavily influence use, stem from different needs, waiting times, and different circumstances.

### Potentially Misguided Judgments of Appropriateness Influence Use

Beyond individual preferences, another key finding was that both patients’ and HCPs’ judgments about when VCs are appropriate influence their use of VCs. However, these judgments did not always align with other research, revealing a tension between personal preference and actual clinical appropriateness.

For instance, patients in this study found VCs useful for initial contact, whereas many HCPs preferred face-to-face meetings for first-time encounters, viewing VCs as more appropriate for follow-ups. This perspective aligns with findings from a Norwegian study, where GPs expressed reluctance to use VCs with new patients [44]. Nevertheless, a systematic review of telehealth use during the COVID-19 pandemic showed that both initial and follow-up visits were commonly conducted via VC, suggesting that in practice, preferences and needs often adapt to context [45]. However, according to HCPs with experience from the pandemic, for some complex issues and with some patients, virtual care had been too time-consuming and had required more effort [37,46]. These concerns, while valid, also raise questions about how much of the perceived burden stems from a lack of support, training, or established workflows for virtual care.

According to both patients and HCPs, the ability to make clinically sound assessments was an important driver of VC use. A study on the transferability of in-person GP consultations to telehealth for patients with diabetes and cardiovascular disease found no clinical tasks that were not transferable [47]. The growing use of home monitoring tools further supports this potential. Yet, limited awareness among HCPs about what can be effectively managed virtually may unnecessarily restrict use.

Health conditions were also seen as factors influencing the use of VCs. HCPs have previously raised usability issues when assessing mental health patients as a reason for preferring face-to-face consultations [9]; however, there seems to be some nuances regarding mental health conditions. According to our findings, VCs are considered suitable for psychosocial issues in general, but some have not deemed VCs suitable for cognitive behavior therapy. Another group of conditions specifically mentioned by HCPs was skin conditions. As 1 HCP expressed, VCs are inappropriate for skin conditions due to poor imaging quality. Canadian HCPs have also reported challenges assessing skin conditions [33]. Appropriateness could depend on the quality of the devices used by patients and HCPs for skin conditions.

To sum it up, both patients’ and HCPs’ judgments of appropriateness influence VC use; however, the line between personal preference and actual clinical appropriateness is slightly blurred.

### Infrastructure, Reimbursement, Sociodemographic, and Organizational Type Impact Use

Beyond preference and judgments of appropriateness, several organizational and societal drivers of VC use were uncovered.

This study highlighted a lack of infrastructure, potentially excluding some patients from using VCs. Restricted access to the necessary eID appears to exclude some patient groups, such as teenagers, a group that also faces barriers in other countries [18]. Language and disability-related barriers have also been attributed to the technology’s shortcomings [18]. Internet connectivity issues, which have previously been described [18,37], can be more challenging in rural areas and contribute to some difficulties during the introduction, influencing substantial use. In addition, reliable systems and proper integration of the systems have been considered essential by HCPs [10].

As previously reported, reimbursement is another factor that influences VC use, both positively and negatively [23]. The reported guilt HCPs feel, in some cases, for charging for VCs highlights a potential issue with the current reimbursement system. There are also reports of work processes influencing use, where nurses might not schedule VCs at certain times. The influence of the work process on VC use has been previously reported [23], and the lack of redevelopment of workflows and routines has been described as being among the everyday challenges of VC implementation [13].

Further, the sociodemographics of the community served by the PCC are another influential factor, according to HCPs. For example, if the infrastructure, that is, eID, is not accessible to most patients, this might influence overall use and impact the HCPs’ perceived usefulness of VCs and intention to use. This could also be further exacerbated by other clinicians’ nonuse [19].

Whether or not the VCs were provided by a private or public organization also influenced patients’ willingness to use the service. The choice between private and public health care has been extensively discussed in the Swedish media and has also been previously reported to influence the intention to use VCs [21]. This could be related to trust and credibility, as eHealth users have expressed this as a necessity, where public actors were considered more credible than private actors [48].

To summarize, suboptimal infrastructure, organizational issues with reimbursement and work processes, as well as sociodemographics and organizational type, impact overall VC use.

### Strengths and Limitations

A strength of this study is its inclusion of both HCPs and patients, offering a comprehensive view of VC use. The diversity among participants, including HCPs from various professions and centers, representing different socioeconomic areas, and patients with different characteristics and VC experiences, adds to this study’s robustness.

However, the youngest patient user group was not included in this study, which is a limitation. As digital natives, younger populations may have unique perspectives on VCs, including different levels of digital literacy, preferences for communication modalities, and expectations of telehealth services. Their absence limits our ability to generalize findings to all patient demographics.

The relatively small sample size may also limit the generalizability of the findings, especially for HCPs, where data saturation was not fully achieved. However, including a diverse sample of HCPs across multiple roles and settings ensured sufficient information power, which was a strength that also reduced the risk of profession-related bias. Moreover, thematic consistency across discussions suggests key insights were captured despite the limited number of focus groups.

Additionally, there is a potential for selection bias, as patients and HCPs who chose to participate might have been more positively inclined toward VCs, potentially skewing the data toward more favorable perceptions. The focus group interview guide was not pilot-tested before data collection, which may have affected the clarity and consistency of the questions; however, we critically assessed the interview guide after each focus group to ensure all important aspects were covered and to improve the clarity of the questions. Furthermore, different tools were used in the data analysis process, both pen and paper and NVivo, which could have introduced inconsistency in the coding and interpretation of the data. Yet, the coding and analysis method used was discussed in detail between the analysts to ensure that the results would not be affected by the different tools used.

As most interviews were conducted remotely, there is a potential loss of details such as body language. However, studies comparing online and face-to-face interviews have identified strengths and weaknesses with both approaches [49], and given that we were interviewing patients and HCPs with experience of VCs, we deemed the method appropriate in this case.

A potential limitation of this study is recall bias, as interviews were conducted 2 years after the implementation of VCs. Participants’ recollections may have been influenced by more recent experiences, making it difficult to capture initial reactions or challenges with complete accuracy. This may have led to an emphasis on enduring issues rather than transient concerns experienced during the early adoption phase. However, by including both HCPs and patients with varying degrees of VC experience, we aimed to mitigate this effect and capture a balanced perspective on long-term usability and adoption.

### Implications and Future Work

Our findings have several key implications for stakeholders involved in the development and implementation of VCs in health care.

For developers of VC applications, this study highlights the importance of optimal usability to promote successful introduction and long-term use. Developers should prioritize extensive usability testing with both HCPs and patients before launch, ensuring the interface is intuitive and accessible to different user groups, such as older adults and people with disabilities. Continuous improvement based on user feedback is essential.

For implementers, a successful introduction of VCs requires multiple strategies. Patients’ concerns need to be understood and addressed. Their digital literacy and self-efficacy also need improvement. Providing multilingual guidance, video tutorials, and accessible materials for nonnative speakers, individuals with visual impairment, and older adults is vital. As key actors in the implementation of VC, HCPs need to be better prepared, adequately trained, and supported. Educational and informational materials developed by physicians have been perceived as useful for clinics lacking prior experience and could be a potential solution to issues HCPs face [18]. Practical information regarding the guidance of people speaking other languages, those with visual impairment, and older people might also be beneficial. Additionally, training programs should help HCPs address biases and assumptions that could hinder adoption, particularly among vulnerable populations. Further, for filling the knowledge gap regarding the appropriateness of VCs and by being in discussion with both patients and HCPs, formulating clear guidelines may be key to fostering patient trust and expanding equitable use. Implementers should also assess and adjust workflows within health care organizations to minimize disruptions to service use.

Health care providers could consider the potential for VCs as a solution to some of the seasonal-related care delivery issues when resource planning.

For policymakers, this study emphasizes the importance of digital readiness, including reliable internet access and the necessary tools such as eIDs. Policymakers should invest in upgrading digital infrastructure to provide universal access, particularly in underserved areas. Additionally, supportive reimbursement systems for VCs should be established to ensure appropriate use, along with long-term investments in health care systems to reduce waiting time and improve service delivery. Enhancing health care providers’ ability to implement new digital interventions is also crucial for building resilience in the face of future crises.

Further research is needed to evaluate the effectiveness of different strategies for introducing patients to VCs, with a focus on addressing inequalities. Studies should also explore younger patients’ perspectives, as their digital habits and preferences may influence adoption. Additionally, more research is required to understand how the characteristics of HCP users and nonusers affect VC usage. Future research could also explore differences in drivers of use across professional roles and between consultations with new vs known patients. Further, to better understand nonuse, future research should involve nonusers to gain their perspectives.

### Conclusions

The pandemic facilitated the introduction of VCs and accelerated use. However, the rushed implementation of VC contributed to a challenging start, potentially deterring some patients and HCPs from use. HCPs played a vital role in the introductions and influenced VC use both positively and negatively. They were unprepared, which contributed to many challenges, and their misguided preconceptions risk reinforcing existing disparities and contributing to digital exclusion. However, patients also relied heavily on HCPs’ recommendations when deciding to accept a VC. Moreover, patients’ biases against themselves, reinforced by initial introduction challenges and broader concerns about fairness, may also influence VC use. In addition, HCPs’ and patients’ preferences, which were related to their needs, waiting times, and different circumstances, influence use. Further, judgments of appropriateness that also influenced use may be misguided. Lastly, infrastructure, reimbursement, sociodemographics, and organizational type were also uncovered as drivers of use.

Based on these findings, developers of VC applications should optimize usability, and implementers should include multiple strategies, including training programs, guidance, and assessing and adjusting workflows. Health care providers should consider the potential of VCs as a solution to some care-delivery issues when planning resources. Additionally, policymakers should invest in digital readiness, including upgrading digital infrastructure, creating supportive reimbursement systems, and long-term investments in health care systems. Further research should evaluate the effectiveness of different strategies for introducing patients to VCs, explore younger patients’ perspectives, characteristics of HCP users and nonusers, differences between professional roles, as well as between consultation types. Nonusers’ perspectives should also be explored.

## Abbreviations

COREQ: Consolidated Criteria for Reporting Qualitative Research
eID: electronic identification
ePrIm: implementation of eHealth in primary care
GP: general practitioner
HCP: health care professional
PCC: primary care center
RAP: rapid assessment procedure
TC: telephone consultation
VC: video consultation
